# Impact of Fluorinated Ionic Liquids on Human Phenylalanine Hydroxylase—A Potential Drug Delivery System

**DOI:** 10.3390/nano12060893

**Published:** 2022-03-08

**Authors:** Márcia M. S. Alves, Paula Leandro, Haydyn D. T. Mertens, Ana B. Pereiro, Margarida Archer

**Affiliations:** 1 Instituto de Tecnologia Química e Biológica António Xavier, Universidade Nova de Lisboa (ITQB NOVA), 2780-157 Oeiras, Portugal; marcia.alves@itqb.unl.pt; 2 LAQV, REQUIMTE, Faculdade de Ciências e Tecnologia, Universidade Nova de Lisboa (FCT NOVA), 2829-516 Caparica, Portugal; 3 Research Institute for Medicines (iMed.Ulisboa) and Department of Pharmaceutical Sciences and Medicines, Faculdade de Farmácia, Universidade de Lisboa, 1649-003 Lisbon, Portugal; aleandro@ff.ulisboa.pt; 4 European Molecular Biology Lamboratory (EMBL), Hamburg Unit c/o Deutches Elektronen Synchrotron (DESY), D-22607 Hamburg, Germany; hmertens@embl-hamburg.de

**Keywords:** fluorinated ionic liquids, surface-active ionic liquids, human phenylalanine hydroxylase, phenylketonuria, encapsulation

## Abstract

Phenylketonuria (PKU) is an autosomal recessive disease caused by deficient activity of human phenylalanine hydroxylase (hPAH), which can lead to neurologic impairments in untreated patients. Although some therapies are already available for PKU, these are not without drawbacks. Enzyme-replacement therapy through the delivery of functional hPAH could be a promising strategy. In this work, biophysical methods were used to evaluate the potential of [N_1112(OH)_][C_4_F_9_SO_3_], a biocompatible fluorinated ionic liquid (FIL), as a delivery system of hPAH. The results herein presented show that [N_1112(OH)_][C_4_F_9_SO_3_] spontaneously forms micelles in a solution that can encapsulate hPAH. This FIL has no significant effect on the secondary structure of hPAH and is able to increase its enzymatic activity, despite the negative impact on protein thermostability. The influence of [N_1112(OH)_][C_4_F_9_SO_3_] on the complex oligomerization equilibrium of hPAH was also assessed.

## 1. Introduction

Ionic Liquids (ILs) are salts comprised of an organic cation and inorganic anion, which present melting points close to 373.15 K. These molten salts present several physicochemical properties, such as viscosity, hydrophobicity, density, solubility, toxicity, and biodegradability, which can be easily tailored by modifying the functional group, anion, or cation [1]. Their versatility and tuneability allow targeted chemical structure design for the desired application. Thus, ILs show significant potential in the pharmaceutical field as enhancers of drug solubility, permeation, and stability, and as reaction media for organic and enzyme stabilization reactions, separations, and extractions [2,3,4]. ILs are also able to improve the stability and activity of proteins and enzymes [5,6]. Protein stability in ILs depends on the IL’s structure and composition, protein surface composition and charge, specific anion/cation effects, and the aqueous environment [6,7]. The structural diversity present in both ILs and proteins suggests multiple and complex solvent–protein interactions [7].

IL surface activity can be increased by lengthening the alkyl chain, yielding surface-active ionic liquids (SAILs) [8,9]. SAILs are environmentally friendly surfactants [10], whose hydrophobicity can be tailored by adjusting the length of the alkyl chain, the type of head group, and the counterion. Therefore, the resulting aggregates, such as micelles, can be finely tuned to have the desired structure and dynamics. Because of their ability to enhance drug permeability across biomembranes and to substitute conventional surfactants in drug carriers, SAILs are very desirable to the pharmaceutical industry for protein formulation and delivery [11,12,13]. Furthermore, SAILs can substitute surfactants in protein preparations with commercial applications such as in cosmetics, detergents, and biochemical reactions [14].

Fluorinated ionic liquids (FILs) are SAILs whose alkyl chain contains a fluorous tag comprised of four or more carbon atoms that self-assemble into distinct structures depending on their concentration in solution [15]. These FILs have been shown to be biocompatible and non-toxic in four human cell lines [16] and in aquatic species with different levels of organization [17]. Moreover, the effect of these FILs on model proteins—lysozyme and bovine serum albumin (BSA)—has been evaluated [18,19]. Further studies are needed with therapeutic proteins to gain insights into their interactions with FILs and assess the potential use of these FILs as protein drug carriers.

In this work, we have selected human phenylalanine hydroxylase (hPAH), a potentially therapeutic enzyme with a molecular mass around 210 kDa and a more complex structural and functional mechanism than the previously studied proteins [18,19]. hPAH is a member of the aromatic amino acid hydroxylase family and catalyzes the conversion of L-Phenylalanine (L-Phe) into L-Tyrosine (L-Tyr) in the liver using tetrahydrobiopterin (BH_4_) and dioxygen as cofactors. Interestingly, besides being a substrate, L-Phe is also an allosteric regulator of hPAH. hPAH assembles as a tetramer (dimer of dimers) where each 52 kDa subunit contains three functional and structural domains: (1) the N-terminal regulatory domain; (2) the central catalytic domain, which contains the binding sites for substrate, iron, and cofactors (active site); and (3) the C-terminal oligomerization domain, which mediates the interaction between the two dimers [20,21,22]. Presently, ~1200 different mutations in the gene coding for hPAH (*PAH*) have been Identified that can affect protein expression, folding, catalysis, or regulation and lead to phenylketonuria (PKU). PKU is characterized by an increase in the concentration of circulating L-Phe (hyperphenylalaninemia), leading, in the central nervous system, to high levels of L-Phe and low concentrations of L-Tyr and L-Trp, the precursors of amine neurotransmitters [23]. Due to L-Phe neurotoxicity and neurotransmitters depletion, untreated patients may present severe psycho-motor delays [24]. The current treatments for PKU include lifelong dietary restrictions of L-Phe sources (dietetic approach) and pharmacological therapies such as supplementation with BH_4_ (acting as a pharmacological chaperone) and enzyme substitution therapy with a pegylated form of the non-human enzyme phenylalanine ammonia lyase (PEG-PAL) [21,22,25]. Nevertheless, disadvantages have been described for all the available therapies [23]. The diet is problematic, unpalatable, and needs to be maintained through life, and thus non-compliance during adolescence and adulthood is a common cause of progressive neurodegeneration. Cofactor supplementation is limited to patients carrying BH_4_-responsive genotypes, usually associated with mild *PAH* missense mutations, and immunogenic events have been described in patients undergoing PEG-PAL therapy. Delivery of functional hPAH (enzyme reposition therapy) to patients suffering from PKU would be an alternative and “universal” strategy to treat this disease, covering the full spectrum of *PAH* genotypes and avoiding the immunogenicity associated with the administration of a non-human enzyme.

Herein, we studied the interaction between hPAH and a biocompatible, choline-based SAIL, [N_1112(OH)_][C_4_F_9_SO_3_], so proof of concept can be established to further develop a novel drug delivery system. Biophysical techniques have been used to assess the impact of this surfactant’s IL on the activity, stability, and structure of hPAH, such as nano differential scanning fluorimetry (DSF), limited proteolysis, circular dichroism (CD), and small-angle X-ray scattering (SAXS). Furthermore, transmission electron microscopy (TEM) and dynamic light scattering (DLS) were used to evaluate protein encapsulation. This work aims to determine whether hPAH remains active, maintaining the necessary preactivation by the substrate after encapsulation by non-toxic, biocompatible [N_1112(OH)_][C_4_F_9_SO_3_], supporting its use for biomedical applications.

## 2. Materials and Methods

### 2.1. hPAH Production

Recombinant full-length hPAH was produced in *Escherichia coli* Top10 cells transformed with pTrcHis-hPAH expression construct. hPAH is produced as a fusion protein with an N-terminal His_6_-tag and a 26 amino acid linker [26]. Bacterial cells were grown in Luria–Bertani broth supplemented with 50 µg·mL^−1^ ampicillin, at 310.15 K and 220 rpm with continuous shaking. When OD_600_ was between 0.4 and 0.6, expression was induced by the addition of 1 mM isopropylthio-β-D-galactoside and 0.2 mM ferrous ammonium sulfate (Fe^2+^). After 3 h at 310.15 K, cells were harvested (3000× *g*, 20 min, 277.15 K), and pellets were resuspended in Lysis Buffer (50 mM sodium phosphate pH 7.8, 300 mM NaCl, 10% glycerol) supplemented with 1 mM phenylmethylsulfonyl fluoride, DNaseI, and 1 mg·mL^−1^ lysozyme. Cells were then disrupted by sonication (Branson Sonifier 450D, Fisher Scientific, Waltham, MA, USA) in three cycles of 60 s at 50% amplitude, and the soluble fraction was recovered after centrifugation (13,000× *g*, 40 min, 277.15 K). The first purification step consisted of immobilized metal affinity chromatography. Ni-NTA resin (Qiagen GmbH, Hilden, Germany) was pre-equilibrated in lysis buffer supplemented with 10 mM imidazole, added to the cell lysate, and stirred for 1 h at 277.15 K. The resin was loaded onto a column and washed with increasing concentrations of imidazole (from 20 to 75 mM), and hPAH was eluted at 250 mM imidazole [27]. Size-exclusion chromatography (SEC) was performed using a Superdex 200 Increase 10/300 column (GE Healthcare, Chicage, IL, USA) in 20 mM HEPES pH 7.0, 200 mM NaCl (SEC buffer), at 277.15 K (see Figure S1 of Supplementary Materials (SM)). Isolated tetramers were flash-frozen and kept at 193.15 K until needed. All further assays were performed using SEC buffer unless otherwise stated.

### 2.2. Enzymatic Assays

**Figure 1:**
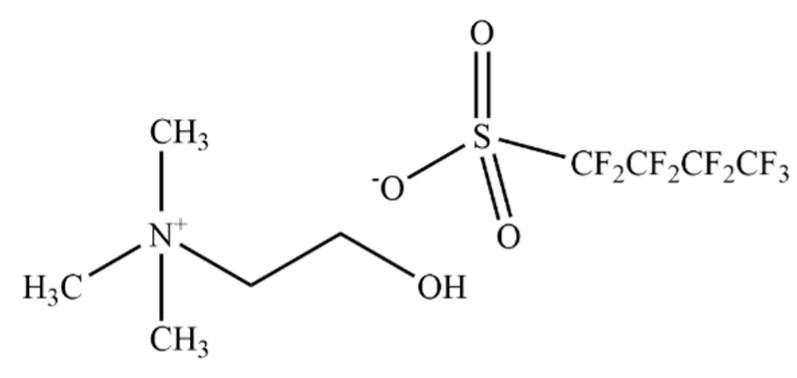
Chemical structure of cholinium perfluorobutanesulfonate, [N_1112(OH)_][C_4_F_9_SO_3_].

The formation of L-Tyr by hPAH was measured as previously described in [28] using a final reaction volume of 200 µL. The reaction mix to follow the activity of hPAH in its preactivated condition was set up using 5 µg His_6_-hPAH, 1 mM L-Phe, 0.1 mg·mL^−1^ catalase, and 0–2% *v/v* cholinium perfluorobutanesulfonate, [N_1112(OH)_][C_4_F_9_SO_3_] (>97% mass fraction purity; Figure 1) supplied by IoLiTec GmbH, Heilbronn, Germany, and incubated for 4 min at 298.15 K. A total of 100 µM Ferrous (Fe^2+^) ammonium sulfate was added and further incubated for 1 min at 298.15 K. The reaction was initiated with 75 µM BH_4_ and stopped after 1 min with the addition of 200 µL of cold 2% (*v*/*v*) acetic acid/ethanol solution. The reaction to study non-activated hPAH activity was set up similarly, but the substrate L-Phe (1 mM) was added simultaneously with BH_4_. The amount of L-Tyr produced was quantified by HPLC using a Symmetry C18 (5 µm) column (Waters Corporation, Milford, MA, USA), a 10% ethanol mobile phase pumped at 1 mL·min^−1^, and fluorimetric detection (λ_exc_ = 274 nm and λ_em_ = 304 nm). Adequate controls were always performed to exclude the presence of possible interferents. Specific activities are presented as the mean ± standard deviation (SD) of triplicate experiments and expressed in nmol of L-Tyr produced during 1 min per mg of hPAH (nmol L-Tyr·min^−1^·mg^−1^).

### 2.3. Nano Differential Scanning Fluorimetry

Experiments of nanoDSF were performed on a Prometheus NT.48 instrument (NanoTemper Technologies GmbH, München, Germany). Protein intrinsic fluorescence was recorded at wavelengths (λ) of 330 and 350 nm as the temperature increased from 293.15 to 363.15 K at a rate of 1 K·min^−1^. Samples of 0.25 mg·mL^−1^ hPAH with increasing concentrations of FIL (from 0 to 2% *v*/*v*) and 1 mM L-Phe were loaded into Prometheus Capillaries (NanoTemper Technologies GmbH, München, Germany). Melting temperatures (*T*_m_) were calculated from the minimum of the first derivative. The *T*_m_ values in the presence of [N_1112(OH)_][C_4_F_9_SO_3_] were compared to those obtained in the absence of FIL to calculate Δ*T*_m_. In addition, to monitor the effect of L-Phe on the stability of the regulatory and catalytic domains, *T*_m_ values in the presence of 1 mM L-Phe were compared with those obtained in its absence (Δ*T*_m_L-Phe) for each condition.

### 2.4. Limited Proteolysis

hPAH was incubated for 10 min at 298.15 K in the absence or presence of 0.6% *v/v* [N_1112(OH)_][C_4_F_9_SO_3_] and/or L-Phe 1 mM. Trypsin was added at 1:200 mass ratio trypsin:hPAH at 298.15 K in SEC Buffer. At each time point, an aliquot was collected, and leupeptin (trypsin:leupeptin mass ratio of 1:1.5) was added to stop the reaction, followed by the addition of 4x loading buffer and sample denaturation at 368.15 K for 5 min. SDS-PAGE was used to assess the proteolytic profile. Samples were loaded into 4–12% Bis-Tris precast gels (Thermo Fisher Scientific, Waltham, MA, USA) and ran with 1× NuPAGE MES Running Buffer (Thermo Fisher Scientific, Waltham, MA, USA) at 190 V. Relative density of the bands was quantified with ImageJ 1.53e (NIH, Bethesda, MD, USA) [29] and fitted to a two-phase exponential decay equation using GraphPad Prism 8 (GraphPad Software Inc., San Diego, CA, USA).

### 2.5. Circular Dichroism

A JASCO J-815 spectropolarimeter (JASCO Inc., Tokyo, Japan) was used to record CD spectra at 293.15 K with a quartz cuvette of 0.1 cm path length. Samples were prepared in 20 mM Tris HCl, 50 mM KCl, pH 7.5, to avoid interference from HEPES buffer with measurements in the far-UV. Samples of hPAH at final concentration of 0.2 mg·mL^−1^ were incubated with 0 to 2% *v/v* of [N_1112(OH)_][C_4_F_9_SO_3_] for at least 10 min at room temperature. CD spectra were acquired from 190 to 260 nm at scan speeds of 50 nm·min^−1^ with 5 accumulations and a response time of 1 s. Spectral deconvolution was carried out with Dichroweb, using the K2D algorithm [30,31,32].

### 2.6. Blue Native Polyacrylamide Gel Electrophoresis (BN-PAGE)

Samples containing hPAH and 0–1.2% *v/v* [N_1112(OH)_][C_4_F_9_SO_3_] were prepared and analyzed using a blue native polyacrylamide gel electrophoresis-based system from Thermo Fisher Scientific (Waltham, MA, USA). The samples were loaded onto a NativePAGE 4–16% Bis-Tris gel with molecular weight marker NativeMark Unstained Protein Standard (Thermo Fisher Scientific, Waltham, MA, USA), and the electrophoresis was run according to manufacturer’s guidelines. Relative band density was analyzed using ImageJ software 1.53e (NIH, Bethesda, MD, USA) [29].

### 2.7. Small Angle X-ray Scattering

SAXS data were collected at beamline P12 operated by EMBL Hamburg at the PETRA III storage ring (DESY, Hamburg, Germany) [33]. Measurements were performed under constant flow in batch mode using a protein concentration of 0.9 mg·mL^−1^ in SEC Buffer, containing 0–1.2% *v/v* [N_1112(OH)_][C_4_F_9_SO_3_] and in the absence and presence of L-Phe (final concentration of 1 mM). Images were recorded using a Pilatus-6M detector at a sample to detector distance of 3.0 m and λ = 0.12 nm, covering the range of momentum transfer 0.01 < s < 7 nm^−1^ (s = 4πsinθ/λ, where 2θ is the scattering angle). Data were processed and analyzed with the ATSAS program suite, version 3.0.3.1 (Hamburg, Germany) [34], using PRIMUS [35] for further subtraction and averaging as required and for radius of gyration (*R*_g_) and other SAXS invariant estimations. The program OLIGOMER [35] (from ATSAS suite) was used for equilibrium analysis of volume fractions of components in solution, with computed scattering intensities of components (PDB ID: 5FII and 2PAH) calculated in FFMAKER (from ATSAS suite) [35].

### 2.8. Dynamic Light Scattering

DLS measurements were performed on a Zetasizer Nano Series ZEN3600 (Malvern Instruments, Malvern, UK) apparatus equipped with a 633 nm laser using a non-invasive back-scattering technique (173°) for detection. A 10% *v/v* stock solution of [N_1112(OH)_][C_4_F_9_SO_3_] in MilliQ water was prepared and filtered prior to use (Millex PVDF filters, 0.22 µM), and hPAH was diluted to final concentration of 0.2 mg·mL^−1^. Samples were equilibrated for 30 min at room temperature prior to measuring. A 30 µL aliquot of each sample was loaded into a Hellma Quartz Cell QS 3.00 mm and measured 3 times. Cell temperature was kept constant at 298.15 K.

### 2.9. Transmission Electron Microscopy

Solutions containing 0.2 mg·mL^−1^ hPAH and 1.2% *v/v* [N_1112(OH)_][C_4_F_9_SO_3_] were pipetted on a 200 mesh copper grid (3 mm diameter) and left to air dry. Excess sample was then removed using filter paper. The grids were imaged at MicroLab-IST (Lisbon, Portugal) in a Hitachi 8100 model with LaB6 filament at a working voltage of 200 kV.

### 2.10. Statistical Analysis

Experimental data were analyzed with GraphPad Prism 8.0 (GraphPad Software, San Diego, CA, USA) using one-way ANOVA followed by Dunnet test when appropriate. The differences were considered significant when *p* < 0.0005.

## 3. Results and Discussion

### 3.1. Stability and Function of hPAH

The activity of hPAH, which measures the conversion of L-Phe into L-Tyr after BH_4_ addition, was 6095 ± 372 nmol Tyr·min^−1^·mg^−1^ upon previous incubation with L-Phe and 1317 ± 117 nmol L-Tyr·min^−1^·mg^−1^ without L-Phe, corresponding to an increase of 4.6× upon L-Phe preactivation (Table 1). As L-Phe is both an allosteric activator and substrate of hPAH, the kinetic behavior of the enzyme depends on the order of addition of the reaction components, where pre-incubation with L-Phe yields higher rates of L-Tyr production [22,36].

**Figure 2:**
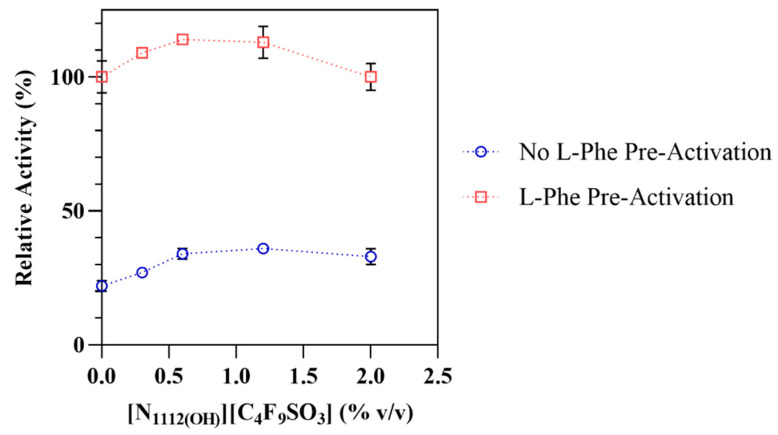
Relative activities of hPAH as a function of [N_1112(OH)_][C_4_F_9_SO_3_] concentrations. Relative enzymatic activity of non-activated and L-Phe activated hPAH. The activity of the hPAH in the absence of [N_1112(OH)_][C_4_F_9_SO_3_] in the preactivated assay (1 mM L-Phe) was considered as 100%. Data are shown as mean ± SD of triplicate assays.

The presence of [N_1112(OH)_][C_4_F_9_SO_3_] at different concentrations, above and below its critical aggregation concentration (CAC) of 0.6% *v/v* [15], shows a growing positive impact in the enzymatic activity of hPAH (see Figure 2 and Table 1), followed by a slight decrease at 2% FIL. The addition of this FIL at 0.6–1.2% *v/v* increased the activity rates of hPAH preactivated with L-Phe up to 13–14%, in contrast to 58–65% for the non-activated enzyme. This pronounced effect suggests that [N_1112(OH)_][C_4_F_9_SO_3_] may preactivate hPAH to some extent in the absence of L-Phe, thus contributing to higher activity rates. As the concentration of [N_1112(OH)_][C_4_F_9_SO_3_] increases, the L-Phe activation ratio decreases, mainly due to the higher hPAH activity in the basal state (non-preactivated).

**Figure 3:**
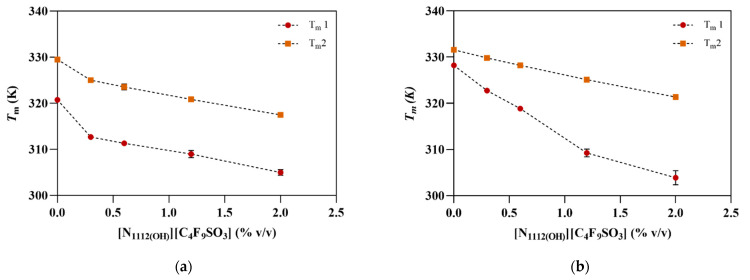
Thermostability of hPAH in the absence and presence of increasing concentrations of [N_1112(OH)_][C_4_F_9_SO_3_]. *T*_m_ values obtained from nanoDSF for hPAH (**a**) in the absence and (**b**) presence of 1 mM L-Phe. Data are shown as mean ± SD of triplicate assays.

The thermal denaturation profile of hPAH, as monitored by nanoDSF, displays two transitions around 320 and 329 K, known to correspond to its regulatory (*T*_m1_) and catalytic (*T*_m2_) domains, respectively (see Figure 3 and Table 2). Pre-incubation with 1 mM L-Phe shifts these transitions to higher temperatures (*T*_m1_ ~328 K, *T*_m2_ ~331 K), thus showing a stabilizing effect (Δ*T*_m_L-Phe: 7.5 and 2 K, respectively), in agreement with previous reports [21,22].

The addition of [N_1112(OH)_][C_4_F_9_SO_3_] to the protein has a detrimental effect on the thermostability of hPAH, which is more significant on the regulatory domain (Δ*T*_m1_ from −8.1 to −15.8 K) than on the catalytic domain (Δ*T*_m2_ from −4.53 to −12.04 K), as can be seen in Figure 3. The presence of L-Phe (1 mM) helps to smooth the protein’s thermal destabilization of the regulatory domain by the FIL up to 0.6% (see Figure S2 of SM), after which Δ*T*_m1_ increases to −18.99 and −24.34 K. The presence of L-Phe also smooths the decrease in *T*_m2_ in the presence of FIL, as Δ*T*_m2_ from −1.75 to −10.23 K were determined. However, when compared to the response of hPAH to L-Phe in the absence of FIL (Δ*T*_m1_L-Phe 7.5 K and Δ*T*_m2_L-Phe 2 K), higher Δ*T*_m1_L-Phe and Δ*T*_m2_L-Phe were observed for FIL concentrations of 0.3 and 0.6% (10.06 and 7.51 K) and 0.3–2% (4.82–3.85 K), respectively.

In the absence of L-Phe, [N_1112(OH)_][C_4_F_9_SO_3_] destabilizes hPAH with a more significant effect on the regulatory domain. This destabilization may leave the catalytic domain more accessible to the substrate, leading to an increase in the activity rate of non-activated hPAH when compared to the control hPAH (0% FIL). The presence of FIL allows the protein to respond to the presence of L-Phe either at the functional and structural level, probably by an effect on the catalytic domain. Indeed, in the presence of different concentrations of FIL, the enzyme activity is 13–14% higher than the control value (6095 nmol Tyr·min^−1^·mg^−1^), correlating with the increase in the Δ*T*_m2_L-Phe observed for the tested concentrations.

**Figure 4:**
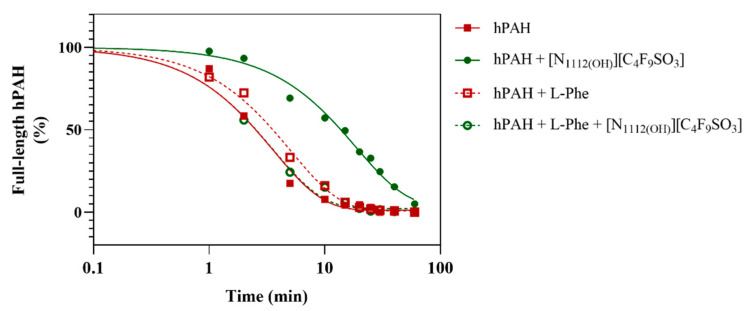
Limited proteolysis of hPAH by trypsin. Degradation of full-length hPAH by trypsin, in the absence and presence of [N_1112(OH)_][C_4_F_9_SO_3_] and/or L-Phe.

hPAH limited proteolysis by trypsin was followed in the presence of 0.6% *v/v* FIL, since hPAH in the preactivated state showed higher enzymatic activity at this concentration (see Figure 2). The presence of [N_1112(OH)_][C_4_F_9_SO_3_] resulted in a 6x slower digestion rate, as demonstrated in Figure 4 and Table 3 (for SDS-PAGE, see Figure S3 of SM). It has been described that decreased proteolytic rates can be due to higher protein compactness or to a lesser mobile protein conformation [22]. In addition, in the specific case of hPAH, higher resistance to proteolysis has also been associated with the movement of the regulatory domain, protecting the C-terminal region of this domain from trypsin digestion and leaving the catalytic domain more accessible to the substrate (high-activity state) [37], which seems to be the case when taking into consideration the activity and thermostability data. In addition, limited access of trypsin to the protein target sequences due to the presence of FIL may be excluded since the addition of L-Phe to the assay leads to a ~5× faster digestion rate. Under these conditions (1 mM L-Phe; 0.6% *v/v* of [N_1112(OH)_][C_4_F_9_SO_3_]), the hPAH proteolytic profile approaches the profile obtained for hPAH without L-Phe and [N_1112(OH)_][C_4_F_9_SO_3_], correlating with the similar *T*_m1_ and *T*_m2_ obtained for these conditions, namely 320.77 ± 0.02 and 329.53 ± 0.03 K (absence of L-Phe and [N_1112(OH)_][C_4_F_9_SO_3_]) and 318.85 ± 0.07 and 328.23 ± 0.02 K (1 mM L-Phe: 0.6% *v/v* [N_1112(OH)_][C_4_F_9_SO_3_]). These data may indicate similar conformational states.

### 3.2. Effect of FIL on hPAH Structure

**Figure 5:**
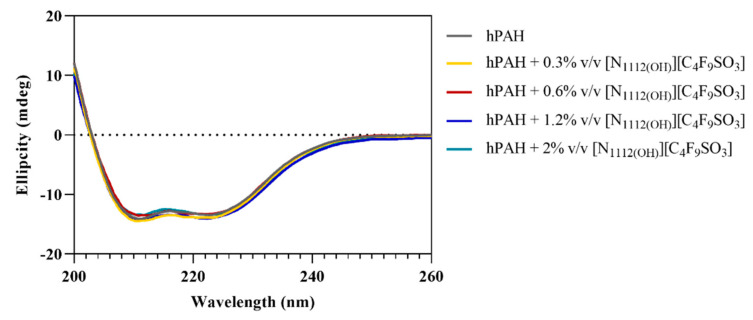
Far-UV CD spectra of hPAH in the absence/presence of [N_1112(OH)_][C_4_F_9_SO_3_] from 0.3 to 2% *v*/*v*.

CD spectrum of hPAH presents two broad minima at 208 and 222 nm (see Figure 5), indicating *α*-helices and *β*-sheets in its secondary structure [21]. The estimated 39% *α*-helical content for hPAH (see Table 4) is in good agreement with previous reports [38]. Upon FIL addition, hPAH maintains its characteristic spectra with small variations in molar ellipticity, indicating that [N_1112(OH)_][C_4_F_9_SO_3_] does not affect the protein’s secondary structure.

**Figure 6:**
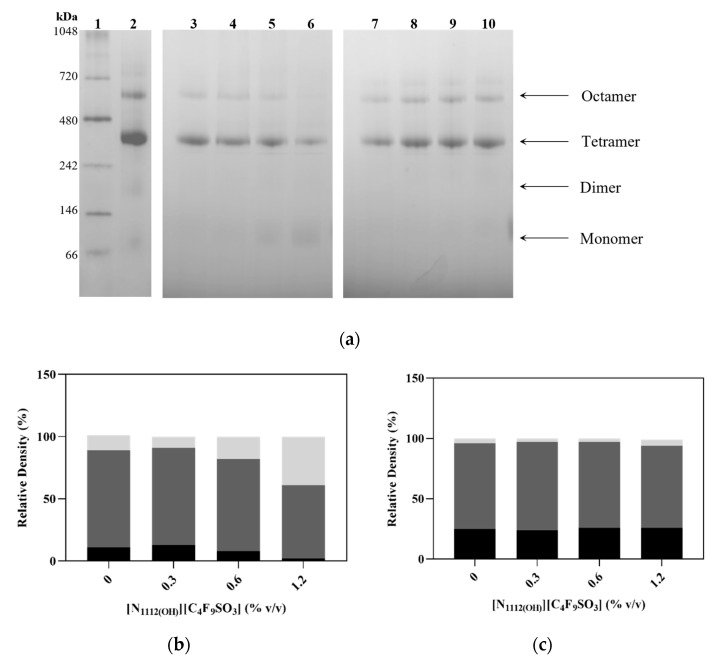

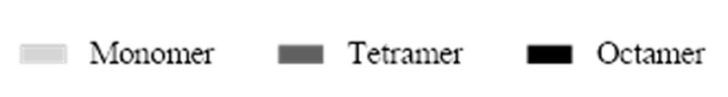
Oligomerization states of hPAH. (**a**) Blue native electrophoresis. Gel 1 (reference): Lane 1–NativeMark; Lane 2—hPAH; Gel 2 (no L-Phe): Lane 3—hPAH, Lane 4—hPAH + 0.3% FIL; Lane 5—hPAH + 0.6% FIL; Lane 6—hPAH + 1.2% FIL; Gel 3 (L-Phe): Lane 7—hPAH + L-Phe, Lane 8—hPAH + L-Phe + 0.3% FIL; Lane 9—hPAH + L-Phe + 0.6% FIL; Lane 10—hPAH + L-Phe + 1.2% FIL. Relative quantification of (**b**) hPAH + FIL and of (**c**) hPAH + L-Phe + FIL calculated using ImageJ software, considering, respectively, Lanes 3 and 7 as controls.

Therefore, the effects observed on the hPAH activity, thermostability, and proteolytic rate are not due to protein denaturation but may result from subtle changes in the tertiary structure of the protein in the presence of FIL. To analyze the impact of [N_1112(OH)_][C_4_F_9_SO_3_] on the quaternary structure of hPAH, blue native electrophoresis was performed (see Figure 6a), as it allows differentiation between the various oligomeric states of hPAH. Tetramers are the predominant form, with some octamers present to a lesser extent (even for the control assay; see Figure 6a, lane 2) as expected [36]. The addition of higher concentrations of FIL (1.2%) leads to a slight increase in monomers, resulting from the dissociation of octamers and tetramers (see Figure 6b). In the presence of L-Phe, a higher level of octamers was found. However, the octameric form of hPAH is maintained, as are the other oligomeric forms. Thus, [N_1112(OH)_][C_4_F_9_SO_3_] (at 0.3 and 0.6% *v*/*v*) does not influence the oligomeric state of non-activated and L-Phe activated hPAH, nor does it promote the formation of aggregates with/without L-Phe.

**Figure 7:**
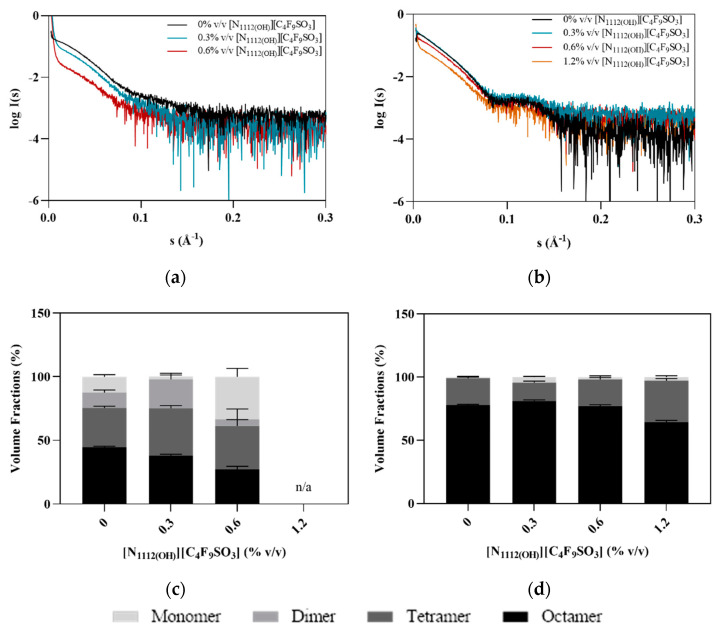
Effect of [N_1112(OH)_][C_4_F_9_SO_3_] on the SAXS profile of hPAH. (**a**) Experimental scattering curves of hPAH + FIL; (**b**) experimental scattering curves of hPAH + L-Phe + FIL; (**c**) volume fractions of oligomers in hPAH + FIL samples; (**d**) volume fractions of oligomers in hPAH + L-Phe + FIL samples.

SAXS data were collected in order to assess possible [N_1112(OH)_][C_4_F_9_SO_3_] induced conformational changes in hPAH. The scattering curves are clearly different in the absence and presence of L-Phe, with the latter showing a broad peak at 0.12 Å^−1^ characteristic of a core–shell-like structure (Figure 7a). This reflects the L-Phe induced assembly of hPAH into a tetrameric and/or octameric structure. In the absence of L-Phe, this peak is absent, suggesting the sample is a polydisperse mixture of different assembly states (e.g., octamers, tetramers, and monomers). In the presence of FIL, the shift between hPAH’s resting (absence of L-Phe) and active state characteristic of domain assembly/rearrangement is maintained, confirming that [N_1112(OH)_][C_4_F_9_SO_3_] does not interfere with the mechanism of hPAH allosteric activation by L-Phe [22,28].

In the absence of L-Phe, evidence of non-specific aggregate formation is observed at low angles, which increases with higher concentrations of FIL. In the presence of L-Phe, however, this behavior is only observed at FIL concentrations above 0.6% *v*/*v*. Furthermore, an oligomerization analysis (see Figure 7b) of the scattering data, using the high-resolution domain structures of hPAH (PDB ID: 5FII, 2PAH) to define association/dissociation components, also suggests the presence of larger species, even before the addition of FIL. This effect has been reported in previous SAXS data collection of hPAH, where the protein tends to equilibrate between different oligomeric states [28,36]. SEC coupled to SAXS (SEC-SAXS) can usually overcome this; however, the interaction between ionic liquids and column matrices precluded the use of the SEC-SAXS mode in this case [39]. The oligomeric equilibrium analysis suggests that the SAXS data in the absence of L-Phe are best fit by a mixture of octameric, tetrameric, dimeric, and monomeric hPAH (Figure 7c,d). The concentration of dimeric hPAH is maintained, while the volume fraction of octameric hPAH decreases with increasing concentrations of [N_1112(OH)_][C_4_F_9_SO_3_] accompanied by an increase in the monomer. For hPAH incubated with L-Phe, the octameric species is dominant, and the dimer is not selected. Interestingly, the ratio of oligomeric species is maintained across the FIL concentration series. These results are in agreement with the blue native electrophoresis described above, confirming that the addition of FIL at the tested concentrations does not influence the oligomerization of hPAH + L-Phe, while for the non-activated protein, a different oligomeric profile was only observed with 0.6% [N_1112(OH)_][C_4_F_9_SO_3_]. Structural parameters such as radius of gyration (*R*_g_), Porod volume (*V*_P_), and approximated average molecular mass (MM), described in Table 5, are in agreement with published hPAH batch (i.e., non-SEC) measurements describing a mixture of oligomeric states [28]. These parameters tend to decrease with increasing FIL concentrations, indicating higher protein compactness on average or the dissociation of larger assemblies into tetrameric/dimeric and monomeric forms. For non-activated hPAH + 0.6% *v/v* FIL, for instance, the calculated MM is 77 kDa, which corresponds to the expected MM of monomeric hPAH [40], the most prevalent species in the sample (Figure 7c). Taking into consideration that octamers were the most prevalent forms for the preactivated hPAH in the presence of IL (Figure 7d) and that a decrease in all structural parameters was observed, the data seem to indicate higher protein compactness for those species as the [N_1112(OH)_][C_4_F_9_SO_3_] concentration increases.

### 3.3. hPAH Encapsulation by FIL

**Figure 8:**
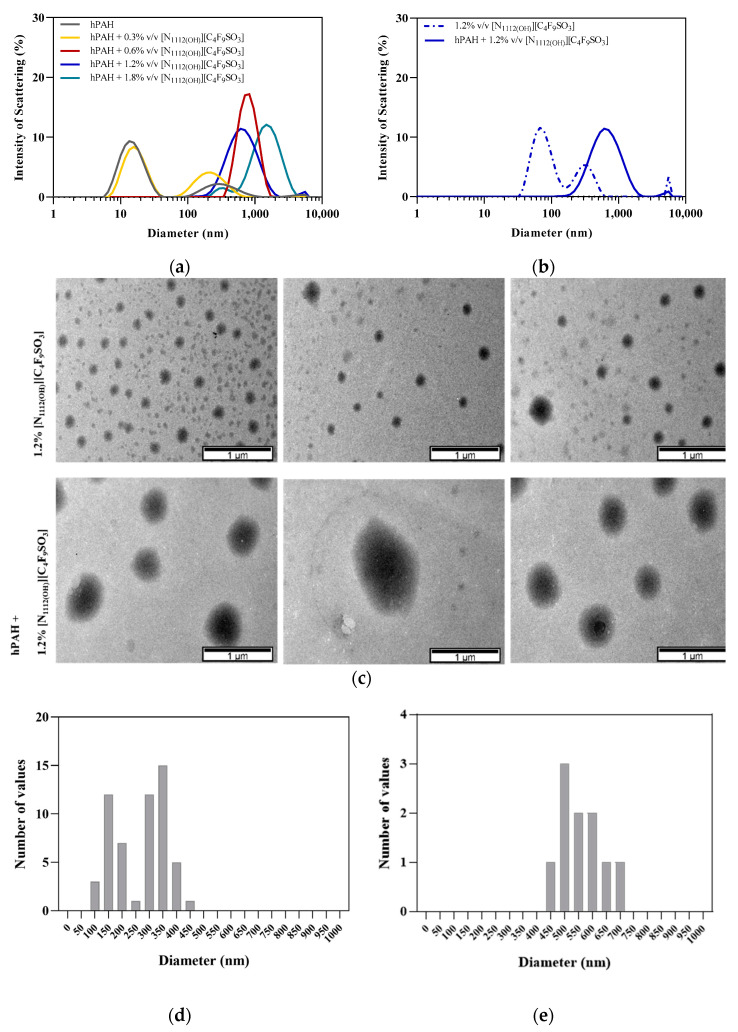
Encapsulation of hPAH by [N_1112(OH)_][C_4_F_9_SO_3_]. DLS spectra of hPAH upon: (**a**) the addition of increasing concentrations of FIL; and (**b**) comparison between 1.2% *v/v* FIL Blank with hPAH + 1.2% *v/v* FIL. (**c**) TEM analysis of 1.2% *v/v* FIL (top) and hPAH + 1.2% *v/v* FIL (bottom). Size distribution (Feret diameter) of particles present in TEM images: (**d**) 1.2% *v/v* FIL and (**e**) hPAH + 1.2% FIL *v*/*v*, quantified using ImageJ software.

In order to assess encapsulation of hPAH by [N_1112(OH)_][C_4_F_9_SO_3_], both DLS and TEM were used to characterize and visualize micelles in solution. The DLS spectra showed a characteristic peak of hPAH at ~14 nm (see Figure 8a) [41], while the spectrum of 1.2% *v/v* [N_1112(OH)_][C_4_F_9_SO_3_] (without hPAH) did not exhibit any aggregates in this range (see Figure 8b), with a bimodal distribution at higher diameter ranges (around 60 and 300 nm). The addition of 0.3% *v/v* FIL led to a decrease in the intensity of the protein peak, although it remains in the solution. At FIL concentrations above CAC (0.6–1.8% *v*/*v*), the protein peak completely disappears, and a single peak appears due to the strong interaction/association of FIL with hPAH, suggesting protein encapsulation. For example, hPAH + 1.2% [N_1112(OH)_][C_4_F_9_SO_3_] exhibits only one broad peak with a mean size of ~600 nm (see Figure 8b). These results show a shift towards higher hydrodynamic diameters for [N_1112(OH)_][C_4_F_9_SO_3_] concentrations > 0.6% *v*/*v*, suggesting encapsulation of hPAH for FIL concentrations above CAC. Similar behavior has already been reported for lysozyme [18].

TEM images were collected in the presence of 1.2% *v/v* [N_1112(OH)_][C_4_F_9_SO_3_], which corresponds to 2× CAC, to maximize the probability of protein encapsulation (see Figure 8c). Control samples (FIL, no hPAH) revealed the formation of heterogeneous micelles, which also displayed a bimodal distribution of particle sizes of approximately 150 and 350 nm (see Figure 8d). This behavior and size distribution was previously observed for similar FILs [18]. In the presence of hPAH, there was a clear increase in particle size to 500–600 nm (see Figure 8e). The sizes measured using the TEM micrographs are in accordance with the diameters obtained by DLS. Therefore, we conclude that, in the presence of hPAH, FIL aggregates become larger and more homogenous due to protein encapsulation. This encapsulation evidence observed by DLS and TEM is corroborated by SAXS. This experimental technique shows some evidence of those aggregates (e.g., upturns in the data at low angles). However, the Dmax of those large aggregates cannot be extracted because huge particles provide scattering intensities that are mostly intercepted by the beamstop. Furthermore, hPAH alone shows a DLS peak that matches reasonably with the SAXS (*D_max_* ~17 nm), confirming the DLS data.

## 4. Conclusions

This study showed that the addition of [N_1112(OH)_][C_4_F_9_SO_3_] at 0.6–1.2% *v/v* to hPAH increased the enzymatic activity in the absence or presence of L-Phe, despite decreasing protein thermostability (nanoDSF). The presence of FIL seems to confer hPAH some protection against trypsin digestion, so we postulate that [N_1112(OH)_][C_4_F_9_SO_3_] may induce structural rearrangements on the N-terminal regulatory domain of hPAH, resulting in a more exposed catalytic domain, with higher basal activity (absence of L-Phe) but lower *T*_m_. Moreover, [N_1112(OH)_][C_4_F_9_SO_3_] does not affect hPAH’s secondary structure (CD) nor its oligomerization states when the protein is pre-incubated with L-Phe; however, in the absence of L-Phe and at 1.2%, it favors the dissociation of octamers/tetramers into monomers (BN-PAGE). [N_1112(OH)_][C_4_F_9_SO_3_] does not contribute to the aggregation of hPAH (BN-PAGE and SAXS). These are promising results as aggregates may lead to loss of function and immunogenic reactions, so this phenomenon should be prevented during protein formulation. In addition, incubation of hPAH with [N_1112(OH)_][C_4_F_9_SO_3_] at concentrations ≥ 0.6% leads to the formation of larger molecular mass species ~1 µm (DLS), and the addition of hPAH to FIL self-assembled particles at 1.2% *v/v* also increases their sizes to similar values (TEM), thus suggesting protein encapsulation. Indeed, [N_1112(OH)_][C_4_F_9_SO_3_] has high surface activity and has been previously shown to be able to form micelles and encapsulate lysozyme [15,18]. The results herein presented show that this FIL is still able to encapsulate a larger and more complex protein while still retaining its structure and function. Although [N_1112(OH)_][C_4_F_9_SO_3_] has been found to be non-toxic in four human cell lines [16] and its eco-toxicity has also been assessed [17], studies on subjects such as the immunogenicity and delivery of this potential drug delivery system are needed to better understand hPAH-[N_1112(OH)_][C_4_F_9_SO_3_] interactions.

## Figures and Tables

**Table 1:** Catalytic activity of non-activated and preactivated hPAH in the absence and presence of increasing concentrations of [N_1112(OH)_][C_4_F_9_SO_3_]. Data are shown as mean ± SD of triplicate assays. (*) Significant statistical differences.

[N_1112(OH)_][C_4_F_9_SO_3_] (% *v*/*v*)	hPAH Activity (nmol Tyr·min^−1^·mg^−1^)		L-Phe Activation Ratio
	L-Phe Preactivation (1 mM L-Phe)	No L-Phe Preactivation	
0	6095 ± 372	1317 ± 117	4.6
0.3	6643 ± 14	1643 ± 28 *	4.0
0.6	6966 ± 60 *	2075 ± 131 *	3.4
1.2	6861 ± 339 *	2178 ± 43 *	3.2
2	6104 ± 298	2011 ± 176 *	3.0

**Table 2:** Variation of hPAH melting temperatures (*T*_m_) measured by nanoDSF. Δ*T*_m_–*T*_m_ variation in the presence of [N_1112(OH)_][C_4_F_9_SO_3_] compared to those in its absence; Δ*T*_m_L-Phe–*T*_m_ variation in the presence versus absence of 1 mM L-Phe.

[N_1112(OH)_][C_4_F_9_SO_3_] (% *v*/*v*)	Δ*T*_m_ (-L-Phe)		Δ*T*_m_ (+L-Phe)		Δ*T*_m_L-Phe	
	Δ*T*_m1_	Δ*T*_m2_	Δ*T*_m1_	Δ*T*_m2_	Δ*T*_m1_ L-Phe	Δ*T*_m2_ L-Phe
0	-	-	-	-	7.5	2.0
0.3	−8.1	−4.5	−5.5	−1.8	10.1	4.8
0.6	−9.4	−6.0	−9.4	−3.3	7.5	4.7
1.2	−11.8	−8.7	−19.0	−6.5	0.25	4.3
2	−15.8	−12.0	−24.3	−10.2	−1.1	3.8

**Table 3:** Effect of L-Phe on the proteolytic stability of hPAH in the presence and absence of [N_1112(OH)_][C_4_F_9_SO_3_] and L-Phe. Proteolytic rates (k_obs_) were obtained from limited trypsin proteolysis.

	*K*_obs_ (min^−1^)	
[N_1112(OH)_][C_4_F_9_SO_3_] (% *v*/*v*)	No L-Phe	1 mM L-Phe
0	0.279	0.197
0.6	0.053	0.281

**Table 4:** Estimation of secondary structure content of hPAH at different concentrations of [N_1112(OH)_][C_4_F_9_SO_3_].

[N_1112(OH)_][C_4_F_9_SO_3_] (% *v*/*v*)	*α*-Helix	*β*-Sheet	*Random coil*
0	0.39	0.09	0.52
0.3	0.40	0.14	0.46
0.6	0.38	0.08	0.54
1.2	0.40	0.11	0.49
2	0.38	0.07	0.55

**Table 5:** SAXS structural parameters of hPAH in the presence and absence of [N_1112(OH)_][C_4_F_9_SO_3_] and L-Phe. Radii of gyration (*R*_g_) were estimated from the Guinier approximation. Excluded particle volumes (*V*_P_) were estimated from the Porod approximation. Maximum particle dimensions (*D*_max_) were obtained from the pair-distribution function. Molecular mass (MM) values were derived from the Porod volume as MM = VP/1.6.

[N_1112(OH)_][C_4_F_9_SO_3_] (% *v*/*v*)	No L-Phe				1 mM L-Phe			
	*R*_g_ (Å)	*V*_P_ (Å^3^)	*D_max_* (Å)	MM (kDa)	*R*_g_ (Å)	*V*_P_ (Å^3^)	*D_max_* (Å)	MM (kDa)
0	53.4 ± 1.1	459,446	168	287	61.7 ± 5.3	741,198	187	463
0.3	50.4 ± 1.2	354,904	137	221	56.7 ± 2.9	617,827	164	386
0.6	46.3 ± 1.8	122,885	117	77	58.7 ± 3.6	556,597	158	348
1.2	-	-	-	-	58.9 ± 4.3	476,911	143	298

## Supplementary Materials

The following supporting information can be downloaded at: https://www.mdpi.com/article/10.3390/nano12060893/s1, Figure S1: Size exclusion chromatography (SEC) profile of human phenylalanine hydroxylase (hPAH), Figure S2: Nano Differential Scanning Fluorimetry raw data, Figure S3: SDS-PAGE analysis of hPAH-limited proteolysis.
