# Supplementary material for: Impact of Fluorinated Ionic Liquids on Human Phenylalanine Hydroxylase—A Potential Drug Delivery System
Source: Nanomaterials (Basel). 2022 Mar 8;12(6):893. doi: 10.3390/nano12060893 (PMC8950220; doi:10.3390/nano12060893)
Supplement: Supplementary file 1 [file nanomaterials-12-00893-s001.zip › nanomaterials-1574516-supplementary.pdf]

nanomaterials

MDPI

Supplementary Materials

Impact of Fluorinated Ionic Liquids on Human Phenylalanine Hydroxylase—A Potential Drug Delivery System

Márcia M. S. Alves [1,4], Paula Leandro [2], Haydyn D. T. Mertens [3], Ana B. Pereiro [4,*] and Margarida Archer [1,*]

[1] Instituto de Tecnologia Química e Biológica António Xavier, Universidade Nova de Lisboa (ITQB NOVA), 2780-157 Oeiras, Portugal; marcia.alves@itqb.unl.pt

[2] Research Institute for Medicines (iMed.Ulisboa) and Department of Pharmaceutical Sciences and Medicines, Faculdade de Farmácia, Universidade de Lisboa, 1649-003 Lisbon, Portugal; aleandro@ff.ulisboa.pt

[3] European Molecular Biology Lamboratory (EMBL), Hamburg Unit c/o Deutches Elektronen Synchrotron (DESY), D-22607 Hamburg, Germany; hmertens@embl-hamburg.de

[4] LAQV, REQUIMTE, Faculdade de Ciências e Tecnologia, Universidade Nova de Lisboa (FCT NOVA), 2829-516 Caparica, Portugal.

* Correspondence: anab@fct.unl.pt (A.B.P.); archer@itqb.unl.pt (M.A.); Tel.: +351-212948318 (A.B.P.); Tel.: +351-214469747 (M.A.)

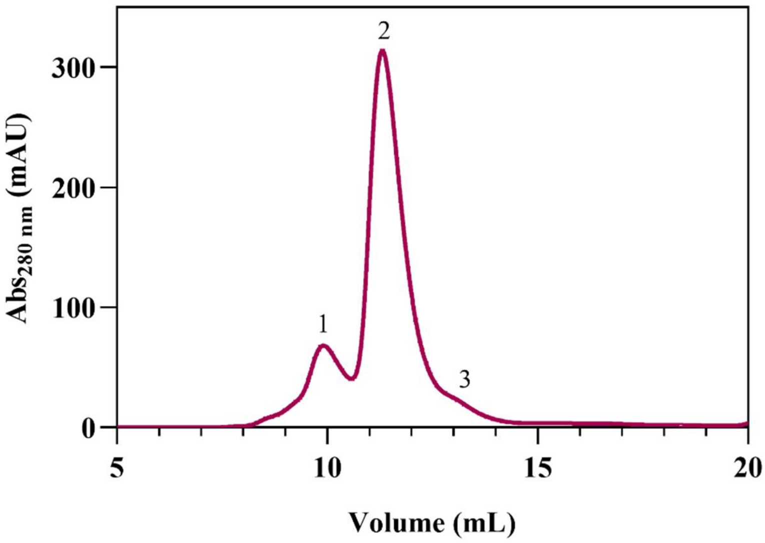

Figure S1. Size exclusion chromatography (SEC) profile of human phenylalanine hydroxylase (hPAH). Peak 1 corresponds to octameric form, peak 2 to tetramer (~220 kDa), and peak 3 to dimer. Apparent molecular masses were estimated using standard molecular masses as reference (not shown). Protein sample collected from peak 2 was used for the assays described in this manuscript.

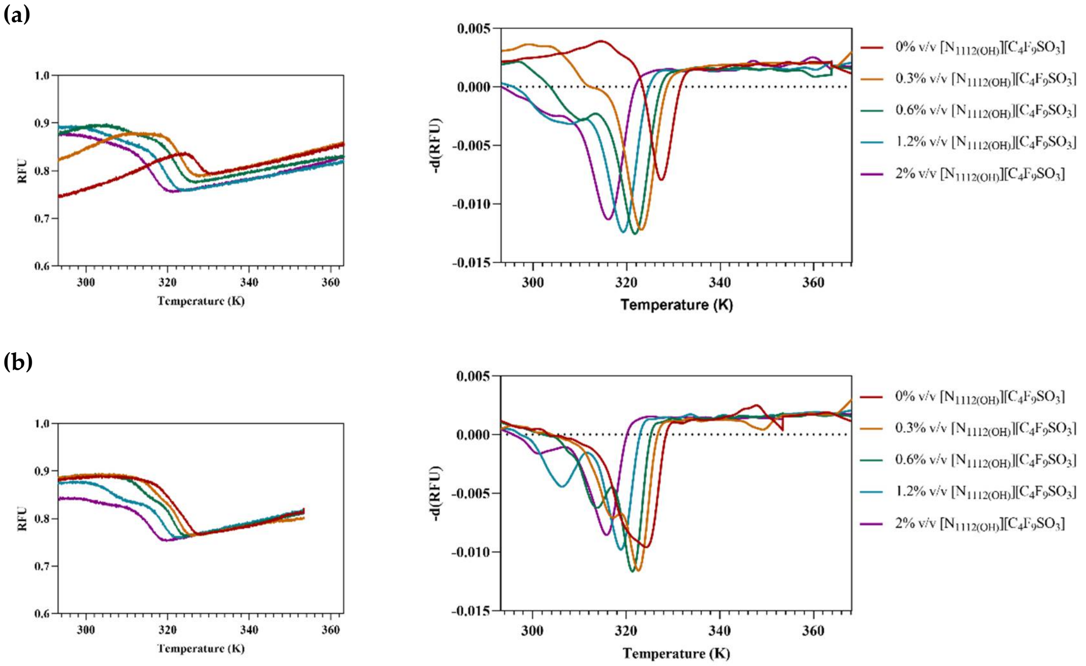

Figure S2. Nano Differential Scanning Fluorimetry spectra *(left)* and first derivative *(right)* for hPAH + 0 – 2% v/v $[N_{1112(OH)}][C_4F_9SO_3]$ in the **(a)** absence or **(b)** presence of 1 mM L-Phe. Data represent the mean of three independent experiments, and error bars are not shown for clarity.

(a)

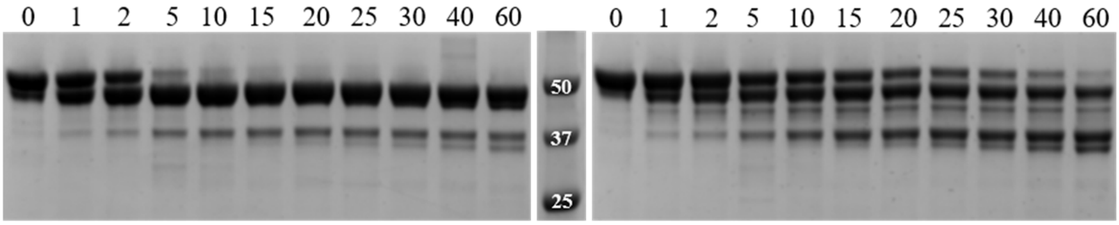

(b)

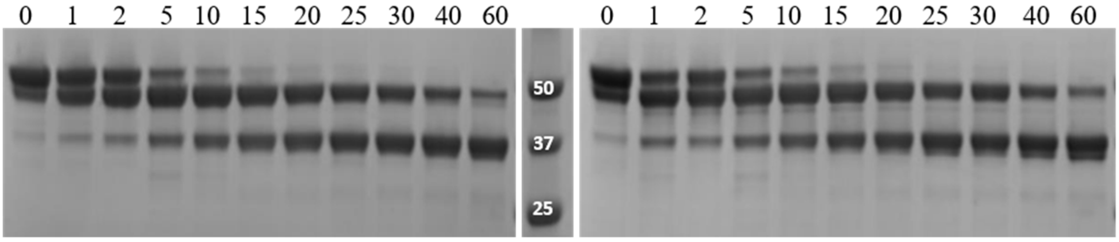

Figure S3. SDS-PAGE analysis of hPAH limited proteolysis. Trypsin was added at 1:200 mass ratio trypsin:hPAH and aliquots were withdrawn at different time points (0 to 60 min). **(a)** hPAH *(left)* and hPAH + 0.6% v/v [$N_{1112(OH)}$][$C_4F_9SO_3$] *(right)*. **(b)** hPAH + 1 mM L-Phe *(left)* and hPAH + 1 mM L-Phe + 0.6% v/v [$N_{1112(OH)}$][$C_4F_9SO_3$]. Each lane corresponds to a time point of the assay, as indicated above the gels (min). Time zero (0) corresponds to an aliquot withdrawn immediately after trypsin addition and some proteolysis is already observed due to the fast removal of the N-terminal domain, and therefore both bands were considered as references for further calculations. The molecular weight marker is found between gels. Gels cropped to highlight bands of interest.
